# Fixation devices for anterior shoulder instability

**DOI:** 10.1186/s40634-023-00610-2

**Published:** 2023-05-04

**Authors:** Lacee K. Collins, Matthew W. Cole, Felix H. Savoie, William F. Sherman, Michael J. O’Brien

**Affiliations:** Department of Orthopaedic Surgery, Tulane University School of Medicine, New Orleans, LA USA

## Abstract

**Purpose:**

Over the past 40 years, advances in the development of anchors and sutures have contributed to the improvement in surgical outcomes for treatment of shoulder instability. Important choices in surgery when treating instability include the use of knotless versus knotted suture anchors, and bony versus soft tissue reconstruction techniques.

**Methods:**

A literature review was conducted to evaluate the history of instability of the shoulder and the results of specific fixation techniques including bony and soft tissue reconstructions as well as knotted and knotless suture anchors.

**Results:**

As knotless suture anchors have continued to grow in popularity since their development in 2001, many studies have compared this newer technique to that of the standard knotted suture anchors. In general, these studies have demonstrated no difference in patient-reported outcome measures between the two options. Additionally, the choice of bony versus soft tissue reconstructions is patient specific as it depends on the specific pathology or combination of injuries.

**Conclusion:**

In each surgery performed for shoulder instability, it is vitally important that we try to restore normal anatomy. The normal anatomy is best established by knotted mattress sutures. However, loop laxity and tear through by the sutures in the capsule can eliminate this restoration, increasing risk of failure. Knotless anchors may allow better soft tissue fixation of the labrum and capsule to the glenoid, but without complete restoration of normal anatomy.

## Introduction

Historically, shoulder instability was addressed surgically with open procedures utilizing drill holes and silk sutures [25]. Over the past 40 years, advances in the development of anchors and sutures have contributed to the ease of surgery and improved outcomes. This opinion article discusses the use of varied fixation devices in the management of shoulder instability.

### History of anterior instability

The most common direction of glenohumeral instability is anterior which makes up greater than 90% of all dislocations [14]. Anterior glenohumeral instability is usually the result of a traumatic injury, and will produce a variety of lesions, including labral and capsule avulsion defects, ligamentous tearing or increased laxity, and glenoid or humeral bone loss [2]. Prior to Perthes and then to Bankart in 1923, recurrent dislocation of the shoulder joint had been attributed to abnormal laxity of the capsule and weakness of the shoulder muscles [4]. However, in his 1923 article Bankart famously described a defect in the anteroinferior glenoid labrum and inferior glenohumeral ligament as a potential cause of recurrent anterior dislocation [4]. Since the introduction of this concept, a multitude of soft tissue and bony procedures have been described for the treatment of anterior glenohumeral instability [14].

### Bony versus soft tissue reconstructions

Bankart described a repair of the lesion utilizing silk suture in a subscapularis tenotomizing approach [4]. In the 1940s, soft tissue transfers were popularized for the treatment of this condition. A procedure described by Gallie and LeMesurier in 1948 utilized a strip of facia lata passed through drill holes in the glenoid, humerus, and coracoid process to reconstruct the deficient structures [15, 25]. Another technique described by Magnuson and Stack in 1943 involves transferring the subscapularis attachment from the lesser tuberosity to the greater tuberosity to increase tension across the anteroinferior joint adding a suspensory element on the humeral head [25, 28]. The Putti-Platt procedure described in 1948 involved dividing the subscapularis tendon and capsule longitudinally and shortening these structures by securing the medial limb to the anterior glenoid and the lateral limb over the top of it [25, 30]. However, both the Magnuson-Slack procedure and the Putti-Platt procedure were associated with decreased external rotation and excessive tightening of the capsule which resulted in progression of glenohumeral arthritis and have since fallen out of favor [19, 25, 27].

Bony defects in the glenoid and/or humerus can also be significant contributors to anterior instability and risk factors for failure of soft tissue repair [8]. A commonly described lesion is the “bony Bankart” which is a detachment of the glenohumeral labral complex with an associated anterior glenoid rim fracture [31]. Humeral head bony defects can also contribute to anterior instability. The typical defect is known as a “Hill-Sachs” lesion which is a compression fracture at the posterolateral portion of the humeral head [21]. The landmark paper by Burkhart and DeBeer revolutionized the assessment of the unstable shoulder, making bone loss evaluation critical in the decision-making process [8]. Itoi, Digiacomo and others developed the concept of the glenoid track, helping us understand the contribution of the defects of both the humerus and glenoid bone to shoulder stability [13]. Numerous treatments have been described to address these associated bony defects. In 1954, Latarjet, unable to perform a Trillat procedure, adjusted and developed fixing the osteotomized coracoid process to augment the glenoid [23]. A similar procedure, described by Helfet and attributed to Bristow, used a single screw to place the coracoid “on end” to increase shoulder stability [20]. In the Latarjet procedure, the transferred coracoid extends the anterior aspect of the glenoid rim thus acting as a bony block [23]. In both the Latarjet and the Birstow procedures, the attached coracobrachialis acts as a sling to increase soft tissue restraints to anterior subluxation. In addition, in both techniques the transferred bone and soft tissue increase the tension on the lower subscapularis, increasing stability [1]. However, the Latarjet and the Bristow procedure may be associated with an increased complication rate as compared to soft tissue repair and mild loss of external rotation [16]. Several treatments of the Hill-Sachs lesion have been described including osteochondral allografts, rotational osteotomies, humeral head resurfacing, and shoulder arthroplasty [3, 11, 17, 40].

In terms of Hill-Sachs lesions, historically the determining factors in whether lesion was surgically addressed was the size and whether it “engages” or not [8]. However, in 2007 Yamamoto et al. introduced the concept of the “glenoid track” and demonstrated that if the Hill-Sachs lesion has a risk of engagement and dislocation if it extends over the medial margin of the glenoid track which can be determined by 3D computed tomography [42]. If this is the case, standard stabilization procedures such as the Bankart repair are unlikely to succeed in isolation [42]. Treatment of Hill-Sachs lesions often involves glenoid bone augmentation such as Latarjet or iliac crest grafting [9, 32]. This prevents engagement of the lesion by lengthening the articular arc of the glenoid [9]. Additional procedures to directly address the Hill-Sachs lesion include remplissage, disimpaction, resurfacing, or arthroplasty. These are typically indicated for Hill-Sachs lesions without concomitant glenoid bone loss [32].

### Knotted versus knotless anchors

**Fig. 1:**
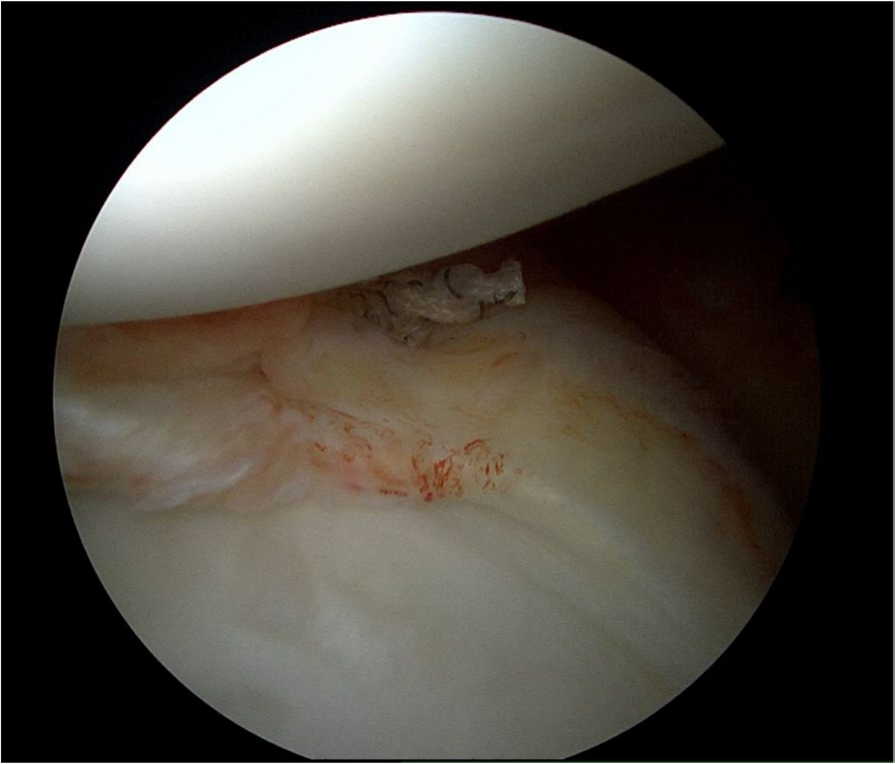
Arthroscopic image of a knotted suture anchor

The initial attempts at arthroscopic Bankart repair involved the use of trans-glenoid drilling, an arthroscopic modification of the Viek technique, as described separately by Caspari, Savoie, and Morgan [10, 29, 34]. The development of the Mitek anchor ushered in the era of suture anchor fixation of the glenoid, avoiding bone tunnels and transglenoid fixation. Anchor development has continued since that time, with metal giving way to plastic then to absorbable and now all suture anchors [39]. The initial anchor sutures were ethibond and quite prone to breakage during attempted knot tying [6]. The development of fiberwire high tensile strength suture was a giant step forward in obtaining more adequate arthroscopic fixation. Secure knot tying has remained elusive for some surgeons, leading to the development of devices that eliminate knot tying due to studies that have shown that the outcome of the repair could be influenced by the knot security of the suture anchor, as well as injuring cartilage surfaces of the glenohumeral joint from the knots [35] (Fig. 1). Additionally, Thal et al. describes that, as arthroscopic repair has become widely used, the technique of knot tying is still inconsistent, yielding lower quality suture knots [37].

**Fig. 2:**
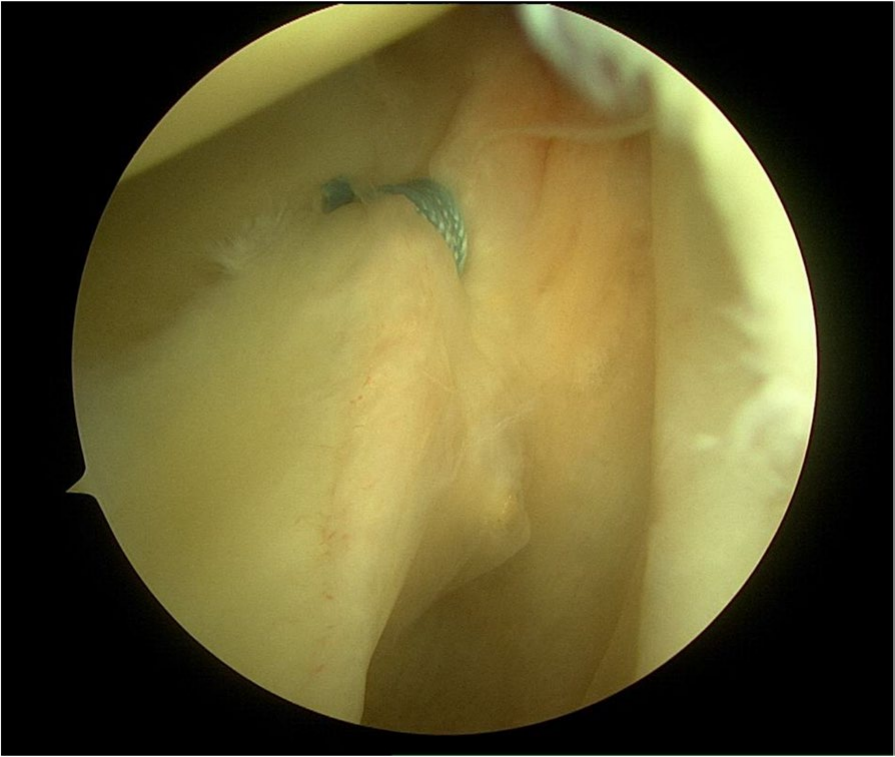
Arthroscopic image of knotless suture anchor

In 1997, Barber et al. restated the knot tying results posed a significant obstacle in arthroscopic surgery [5]. With the inconsistencies in knotted sutures and outcomes in arthroscopic repairs of shoulder instability, new operative techniques emerged to help eliminate or reduce these problems. In 2001, Thal published the first article using knotless suture anchors [38]. These were described to have a short loop of suture secured to the end of the anchor, with a channel, located at the tip of the anchor, functioning to capture the loop of suture [38]. Thal also demonstrated that this new surgical technique provided increased suture strength compared with standard knotted suture anchors [38]. As such, the utilization of knotless sutures proposed a novel method to avoiding the complications and concerns regarding technique and outcome of knotted suture anchors (Fig. 2).

Biomechanical cadaveric studies have recently demonstrated that knotless suture anchors showed similar or greater biomechanical strength to knotted suture anchors [22, 24]. Wu et al. demonstrated that knotless suture anchors had similar rates of re-dislocation and revision surgery, but lower rates or recurrent subluxation, compared to knotted suture anchors in a retrospective study of 102 patients [41]. Additionally, Bents et al. conducted a 1-year follow-up study of 226 repairs using either knotless or knotted suture anchors and demonstrated that no difference in patient-reported outcome measures were found between the cohorts, but that operative time was shorter for patients who received knotless suture anchors [7]. This is consistent with other studies also demonstrating that no differences in activities of daily living or patient reported outcomes are seen between the two options [33, 43]. As such, both the knotless sutures and traditional knotted suture anchors are still currently used in arthroscopic repairs for anterior shoulder instability.

### Where are we going?

In each instability surgery it is vitally important that we try to restore normal anatomy. The labrum is not a bumper, but sits on the face of the glenoid, providing a connection of the more elastic capsule to the more rigid bone. In addition, proper restoration of the labrum re-established the “suction cup” effect which also increases stability [26]. The normal anatomy is best established by knotted mattress sutures [18]. However, loop laxity and tear through by the sutures in the capsule can eliminate this restoration, increasing risk of failure. Changing from mattress to simple sutures, but with knot tying will decrease the suture pull through risk but does not address the loop laxity issue. Knotless anchors, now with tape, may allow better soft tissue fixation of the labrum and capsule to the glenoid, but without complete restoration of normal anatomy [12]. The is also the risk of the exposed suture “rubbing” on the articular cartilage, increasing the risk of arthritis [36].

### Authors recommendations

In the more critical 6 o’clock position we believe double loaded anchors with mattress sutures are needed to begin the capsular shift and restore normal labral anatomy. Similarly, below the equator of the joint we believe knotless anchors provide better anatomical support, as long as the surgeon is comfortable with secure knot fixation techniques. Above 3 o’clock position it is our opinion that knotless anchors seem to provide better fixation and less risk to articular cartilage than in the more inferior position.
